# Fibroblast Growth Factor-21 to Adiponectin Ratio: A Potential Biomarker to Monitor Liver Fat in Children With Obesity

**DOI:** 10.3389/fendo.2020.00654

**Published:** 2020-09-17

**Authors:** Emir Tas, Shasha Bai, Xiawei Ou, Kelly Mercer, Haixia Lin, Kori Mansfield, Robert Buchmann, Eva C. Diaz, Jon Oden, Elisabet Børsheim, Sean H. Adams, Jonathan Dranoff

**Affiliations:** ^1^Department of Pediatrics, University of Arkansas for Medical Sciences, Little Rock, AR, United States; ^2^Endocrinology and Diabetes, Arkansas Children's Hospital, Little Rock, AR, United States; ^3^Arkansas Children's Research Institute, Little Rock, AR, United States; ^4^Arkansas Children's Nutrition Center, Little Rock, AR, United States; ^5^Center for Biostatistics, The Ohio State University Wexner Medical Center, Columbus, OH, United States; ^6^Department of Radiology, University of Arkansas for Medical Sciences, Little Rock, AR, United States; ^7^Department of Medicine, Division of Gastroenterology and Hepatology, University of Arkansas for Medical Sciences, Little Rock, AR, United States

**Keywords:** fibroblast growth factor-21, adiponectin, non-alcoholic fatty liver disease, childhood obesity, intrahepatic triglyceride, magnetic resonace imaging (MRI), leptin

## Abstract

**Background:** There is a pressing need for effective and non-invasive biomarkers to track intrahepatic triglyceride (IHTG) in children at-risk for non-alcoholic fatty liver disease (NAFLD), as standard-of-care reference tools, liver biopsy and magnetic resonance imaging (MRI), are impractical to monitor the course disease.

**Objective:** We aimed to examine the association between serum fibroblast growth factor (FGF)-21 to adiponectin ratio (FAR) and IHTG as assessed by MRI in children with obesity.

**Methods:** Serum FGF21 and adiponectin levels and IHTG were measured at two time points (baseline, 6 months) in obese children enrolled in a clinical weight loss program. The association between percent change in FAR and IHTG at final visit was examined using a multiple linear regression model.

**Results:** At baseline, FAR was higher in the subjects with NAFLD (*n* = 23, 35.8 ± 41.9 pg/ng) than without NAFLD (*n* = 35, 19.8 ± 13.7 pg/ng; *p* = 0.042). Forty-eight subjects completed both visits and were divided into IHTG loss (≥1% reduction than baseline), no change (within ±1% change), and gain (≥1% increase than baseline) groups. At 6 months, the percent change in FAR was different among the three groups (*p* = 0.005). Multiple linear regression showed a positive relationship between percent change in FAR and the final liver fat percent in sex and pubertal stage-similar subjects with NAFLD at baseline (slope coefficient 6.18, 95% CI 1.90–10.47, *P* = 0.007), but not in those without NAFLD.

**Conclusions:** Higher value in percent increase in FAR is positively associated with higher level of IHTG percent value at 6 months in children with baseline NAFLD. FAR could be a potential biomarker to monitor the changes in IHTG in children with NAFLD.

## Introduction

Non-alcoholic fatty liver disease (NAFLD) is the most common etiology of chronic liver disease in children and adults (1, 2). The estimated prevalence of NAFLD is 29 to 38% in children with obesity; however, findings vary among populations studied and diagnostic criteria used (1).

NAFLD represents a disease spectrum, ranging from simple steatosis (i.e., presence of macrovesicular fat in more than 5% of the liver volume) to non-alcoholic steatohepatitis (NASH), cirrhosis, and liver failure (3). The diagnosis of NAFLD requires a demonstration of fatty infiltration on histology or imaging. Obesity is the most important risk factor for the development of steatosis, although in rare instances, individuals with no apparent obesity may also have NAFLD (4). Factors that predict disease progression from steatosis to more advanced stages are not fully understood; however, people with one or more of the components of metabolic syndrome are at a higher risk for adverse outcomes (5). Recently, the degree of hepatic fat accumulation has been hypothesized to play an independent role in the development of NASH and fibrosis (6, 7). This notion was supported by the finding that among adult NAFLD patients with no baseline hepatic fibrosis, those with a higher baseline liver fat (≥15.7%) developed fibrosis at a much higher rate (OR 6.67, 95% CI: 1.01–44.1, *p* < 0.05) after a 1.75 year follow up (8). Moreover, genetic variants that are associated with NAFLD and disease progression, including *PNPLA3* and others, regulate intrahepatic fat trafficking and cause increased hepatic fat accumulation (9). Routine measurements of liver fat, fibrosis, and molecular liver markers to track NAFLD and NAFLD progression are not facile or appropriate for many clinical settings. Thus, identifying biomarkers that can predict static and longitudinal changes of the hepatic fat content and, in particular, the NAFLD phenotype would have prognostic value for disease progression, and serve as a tool to monitor response interventions.

Liver biopsy and magnetic resonance-based imaging techniques are the reference standards for the diagnosis and monitoring of NAFLD (10); however, both have significant limitations for routine use, such as high cost and limited availability (11). Considering the magnitude of NAFLD in the general population, and the limitations of the currently available clinical screening tools (e.g., waist circumference, serum ALT level, liver ultrasonography), recent studies have focused on identifying biomarkers that are effective and cost-efficient for screening, diagnosis and monitoring of NAFLD. Fibroblast Growth Factor-21 (FGF21) has been recognized as an important mediator in hepatic lipid metabolism, and suggested as a biomarker for NAFLD.

Our working hypothesis is that blood analytes associated with whole-body and liver metabolic health (e.g., FGF21 and one of its downstream effectors adiponectin), either singly or in combination, can be used as adjunct predictive tools for NAFLD status and liver fat changes in children. Fibroblast Growth Factor-21 (FGF21) is a distinct metabolic regulator with pleiotropic effects on whole-body energy metabolism (12–14). FGF21 is primarily expressed in the liver and secreted in the circulation in response to starvation/amino acid deprivation and acute exercise (13, 15). An important site of action for FGF21 is adipose tissue, where it increases adiponectin synthesis and secretion (16). Animal and human studies have shown that FGF21 improves peripheral insulin sensitivity, stimulates glucose uptake and lipid oxidation, and decreases lipogenesis, at least in part through increased adiponectin (14, 17–19). Paradoxically, the circulating level of FGF21 is higher, but adiponectin level is lower in individuals with NAFLD, suggesting a state of adipose tissue FGF21 resistance (17, 20–23). Moreover, pharmacological administration of recombinant FGF21 reduced serum triglyceride (TG) and low-density lipoprotein cholesterol (LDL) while increased high-density lipoprotein cholesterol (HDL) levels, and it reversed steatosis in mouse models of obesity and diabetes (14, 17, 24). Humans with obesity and type 2 diabetes had a similar response in serum lipid profile to exogenous FGF21 (25–27). A positive dose-response relationship was observed between the dose of FGF21 administered and the concentration of serum adiponectin in these studies. Therefore, adiponectin levels could potentially reflect, in part, FGF21 actions. With this in mind, we hypothesize that monitoring both hormones has value in terms of biomarker utility in tracking liver fat content in individuals with NAFLD.

This study aims to determine the short-term longitudinal relationship between FGF21, adiponectin and liver fat and to investigate the role of FGF21-Adiponectin Ratio (FAR) as a potential marker to monitor change in liver fat percent (as assessed by MRI) in obese children and adolescents at risk for NAFLD.

## Materials and Methods

### Subjects

Sixty children ages 10–17 years who were seen for weight management at the Center for Obesity And its Consequences in Health (C.O.A.C.H.) clinic at the Arkansas Children's Hospital were recruited. Inclusion criteria included body mass index (BMI) ≥95th percentile for age and sex, all ethnicities, and absence of diabetes (diabetes was defined as fasting glucose ≥126 mg/dL or an HbA1c ≥6.5%). Subjects were excluded from the study if they had a history of cardiac, pulmonary, renal, neurological diseases, and liver disease including autoimmune hepatitis, viral hepatitis, Wilson's disease, hemochromatosis, and biopsy or magnetic resonance imaging-confirmed diagnosis of NAFLD. Also, those taking any prescription medications that are known to have a direct effect on hepatic lipid metabolism (i.e., metformin, statins, fibrates, steroids, thyroid hormones, growth hormones) were excluded.

The Institutional Review Board of the University of Arkansas for Medical Sciences approved the study. Written informed consent and assent were obtained from the legal representatives of each subject and participants, respectively, before participation.

### Study Design

All subjects had a complete medical history and physical exam, including determination of pubertal status by the same Endocrinologist, according to Tanner staging at the baseline visit. Participants in Tanner stage II or III of pubertal development were classified as being in the early stages of puberty, and those in Tanner stage IV or V were classified as being in late stages of puberty. At baseline, each participant received standard of care lifestyle counseling regarding weight management, including guidance about diet and physical activity by a registered dietitian and physical therapist, respectively. Final study visit took place when subjects returned to C.O.A.C.H clinic after 6 months. Adherence to recommendations was not assessed due to the observational nature of the study.

### Blood Analytes

We obtained blood samples by venipuncture (typically from the antecubital vein) after an overnight fast to measure the comprehensive metabolic profile. Serum concentrations of glucose, insulin, triglyceride (TG), high-density lipoprotein (HDL), low-density lipoprotein (LDL), free fatty Acid (FFA), and the level of liver enzymes [alanine aminotransferase (ALT), aspartate aminotransferase (AST), gamma-glutamyl transferase (GGT)] were measured via clinical analyzer (Siemens Atellica, Malvern, PA) at the Arkansas Children's Hospital Chemistry. Serum FGF21 (Human FGF21 Quantikine), adiponectin (Human Total Adiponectin/Acrp30 Quantikine), and leptin (Human Leptin Quantikine) were measured via ELISA per manufacturer's protocols (R&D Systems, Minneapolis, MN). FGF21 to Adiponectin Ratio (FAR) was calculated simply by dividing the FGF21 concentration (pg/mL) to adiponectin concentration (ng/mL). “Percent change” in FAR was calculated by subtracting the baseline value from the final value divided by the baseline value multiplied by 100. A positive number indicates an increase while a negative number indicates a decrease in FAR. Percent change was chosen over absolute change because of the wide interindividual variation in FGF21 concentrations and non-normal distribution of the FGF21 and FAR. Similarly, Leptin to Adiponectin Ratio (LAR) was calculated simply by dividing the leptin concentration (pg/mL) to adiponectin concentration (ng/mL). Homeostatic Model Assessment for Insulin Resistance (HOMA-IR) was calculated by the following formula: fasting glucose (mg/dL) × fasting insulin (mIU/mL) divided by 405.

### Body Composition

Total body adiposity was assessed via bioimpedance technique using InBody® 570 body composition analyzer (InBody USA, Cerritos, CA) according to the manufacturer's protocols. In brief, tissue impedance is measured over 60 s when a low intensity current travels between the bare feet and hands of the subjects. The total body fat (TBF) estimate was obtained from the equipment software. TBF estimate via InBody correlates well with the Dual X-Ray Absorptiometry (DXA) scan (28).

### Liver Magnetic Resonance Imaging

All subjects had an estimation of intrahepatic triglyceride (IHTG) percent by magnetic resonance imaging (MRI) at baseline and final visits. Specifically, a multi-echo multi-slice gradient-echo pulse was used to acquire in/out of phase images of the whole liver using a 1.5T MRI scanner (Philips Healthcare, Best, The Netherlands). MRI method was chosen over conventional magnetic resonance spectroscopy (MRS) because MRI in principle uses the same method (Dixon method) as MRS, but can provide an evaluation of the fat concentration of the whole liver (other than one single imaging voxel by MRS that also has to be manually placed), which would be preferred for this longitudinal study. In addition, studies have shown close agreement between MRI and MRS measurements of fat fraction in children with known or suspected NAFLD (29). The triple-echo method was used to control/reduce the confounding effects of intrinsic T2/T1 relaxation in the liver fat quantification (30, 31).

### Quantification of Intrahepatic Triglyceride (IHTG) Content and Percent

All raw MRI images were exported to a workstation with MATLAB software (The MathWorks Inc., Natick, MA) and customized script for post-processing and for calculation of whole liver average fat concentration. Two experienced raters (XO, KM) sketched a region-of-interests (ROI) for each subject which included the whole liver as much as possible, but avoided intrahepatic vessels and perihepatic fat as well as all edges. The average signal intensity in the selected ROI was computed, and the liver fat concentration for the subject was calculated from these signal intensities.

Subjects were classified as having NAFLD if the liver fat percent by MRI was ≥5% at baseline or final visits. Furthermore, we divided the subjects into three groups based on the “absolute change” in the IHTG percent between the baseline and final studies: (i) subjects whose final liver percent was ≥1% point lower than the baseline (Loss), (ii) subjects whose final liver fat percent was within −1% and +1% point of the baseline (No change), and (iii) subjects whose final liver fat percent was ≥1% point higher than the baseline (Gain). For instance, a subject with 5% IHTG at baseline, with a repeat MRI showing ≤4% liver fat, would be categorized in the loss group. Using this same example, if the repeat MRI shows ≥6% liver fat, then the individual would be categorized in the gain group; however, if the repeat MRI shows liver fat between 4 and 6%, then this individual will be in the no change group. In our study, 1% absolute change in IHTG corresponded to a 46% relative reduction of the liver fat in the loss group and 37% relative increase in the gain group. Patel et al. (32) utilizing paired MRI and liver biopsy data showed that a 29% relative reduction in liver fat was associated with a histological improvement in patients with NASH. As such, it is reasonable to expect the subjects in the loss vs. gain groups in our study to have a clinically relevant amount of improvement vs. worsening in their liver histology, respectively.

### Statistical Analysis

Summary statistics presented are mean (SD, standard deviation) or median (Q1, Q3) for continuous variables, and count (percentages) for categorical variables. Comparative analyses between two groups (loss vs. gain, or NAFLD vs. No NAFLD) were assessed by Wilcoxon Rank-sum test for continuous variables due to small sample size or non-normality, two-sample *t*-test for normally distributed continuous variables, and Fisher's exact test or Chi-square test for categorical variables. Comparative analyses between three groups (loss, no change, and gain) were assessed by Kruskal–Wallis test for continuous variables due to small sample size or non-normality, and Fisher's exact test for categorical variables. Due to the wide variability of FGF21 reported in previous literature, all outlying values outside of the whiskers of the boxplots were retained in the analyses. Sensitivity analyses were conducted using different cut-off values for the definition of loss and gain groups. Finally, a multiple linear regression model predicting IHTG percent at final visit was built using percent change in FAR, baseline NAFLD status, and their interaction, and adjusting for sex and pubertal stage in order to assess the relationship between FAR and liver fat percent. A similar model was also fit using LAR in replacement of FAR. *P* < 0.05 were considered statistically significant. All analyses were implemented in Stata 16.1 (StataCorp, College Station, TX).

## Results

### Subjects' Characteristics

Of the sixty subjects recruited, fifty-eight completed baseline study visit and liver MRI measurements. Twenty-three subjects (39%) had NAFLD at baseline. Ten participants were lost to follow-up (retention rate 48/58 = 83%). Overall, 48 subjects completed both study visits and MRI (baseline and 6-month), and are included in the final comparative analyses. Regardless of the baseline NAFLD status, 13 (27%) subjects had ≥1% reduction in IHTG percent (loss group), while 17 (35%) subjects had ≥1% increase in IHTG percent (gain group) than baseline level. Eighteen (38%) subjects were categorized in the no change group as they had <1% increase or decrease in IHTG percent.

### Clinical and Laboratory Characteristics

Characteristics of subjects with and without NAFLD at baseline were summarized in Table 1. Comparison of the baseline characteristics of all subjects by sex and pubertal stage provided in the Supplementary Tables 1, 2. At baseline mean FAR was significantly higher in the subjects with NAFLD compared to those without NAFLD, while FGF21 and adiponectin levels were not significantly different. The difference in FAR among those with and without NAFLD was independent of total adiposity as serum leptin concentrations and percent body fat were comparable between these two groups. Subjects with NAFLD had significantly higher waist circumference, fasting insulin, HOMA-IR, and ALT levels. Weight, BMI, BMI-z, and LAR were all higher in subjects with NAFLD, but none of them reached statistical significance (Table 1). FAR at baseline was correlated with the IHTG percent on MRI (Spearman correlation coefficient *r* = 0.27, *p* = 0.0397). Among the participants with paired MRI and FAR data (*n* = 47), the percent change in FAR correlated significantly with the percent change in IHTG (Spearman correlation coefficient *r* = 0.53, *p* < 0.001; Supplementary Table 3).

**Table 1:** Summary of all subject characteristics at baseline and comparison among subjects with and without NAFLD.

	**All *N* = 60**	**No NAFLD *N* = 35**	**NAFLD *N* = 23**	***P***
Hepatic fat %	6.2 (5.5)	2.8 (1.1)	11.5 (5.4)	<0.001
Sex, male	26 (43%)	16 (46%)	10 (43%)	0.87
Age, years	14.2 (2.0)	14.1 (1.8)	14.4 (2.3)	0.56
Stage of puberty, advanced	43 (72%)	24 (69%)	17 (74%)	0.66
Ethnicity/Race, Hispanic	16 (27%)	4 (11%)	12 (52%)	0.005
Weight (kg)	101.5 (21.9)	96.9 (19.0)	106.6 (24.7)	0.099
BMI (kg/m^2^)	37.2 (6.3)	35.7 (5.4)	38.8 (7.0)	0.061
BMI, *z*-score	2.4 (0.3)	2.4 (0.3)	2.5 (0.2)	0.061
Total body fat (%)	44.7 (6.4)	43.3 (6.3)	45.8 (6.0)	0.14
Waist circumference (cm)	113.1 (14.5)	108.7 (13.3)	117.7 (13.5)	0.016
SBP (mmHg)	127.8 (10.0)	126.0 (9.3)	129.8 (10.5)	0.15
DBP (mmHg)	70.2 (5.5)	70.3 (5.9)	69.6 (4.8)	0.63
Glucose, fasting (mg/dL)	93.6 (9.4)	95.4 (10.3)	91.4 (7.6)	0.12
Insulin, fasting (uIU/mL)	29.3 (17.6)	23.9 (15.9)	37.0 (17.9)	0.005
HOMA-IR	6.8 (4.2)	5.7 (4.1)	8.4 (4.1)	0.017
FGF-21 (pg/mL)	156.2 (104.3)	140.1 (84.1)	190.3 (123.0)	0.072
Adiponectin (ng/mL)	7.6 (3.6)	8.3 (3.7)	6.7 (3.4)	0.097
Leptin (pg/mL)	60.5 (33.4)	55.5 (27.9)	61.3 (34.7)	0.49
FAR (pg/ng)	25.7 (29.1)	19.8 (13.7)	35.8 (41.9)	0.042
LAR (pg/ng)	10.1 (7.6)	8.1 (5.4)	11.8 (8.7)	0.056
Triglyceride (mg/dL)	109.8 (57.1)	104.9 (65.3)	117.5 (45.3)	0.42
HDL (mg/dL)	43.9 (8.3)	45.2 (8.2)	41.3 (8.0)	0.078
FFA (mmol/L)	4.7 (2.0)	4.7 (2.4)	4.8 (1.4)	0.80
ALT (IU/L)	32.4 (13.8)	29.2 (12.2)	38.3 (14.7)	0.013
AST (IU/L)	28.0 (12.7)	27.9 (14.5)	28.7 (10.1)	0.81
GGT (IU/L)	24.4 (10.5)	22.1 (9.8)	27.3 (11.2)	0.066

*Statistics shown are N (%) or median (Q1, Q3). P-values are calculated from Fisher's Exact test or Chi-square test for categorical variables, and two-sample t-test for continuous varibales. ALT, alanine aminotransferase; AST, aspartate aminotransferases; BMI, body mass index; DBP, Diastolic Blood Pressure; FAR, Fibroblast Growth Factor 21–Adiponectin Ratio; FFA, free fatty acid; FGF21, fibroblast growth factor 21; GGT, Gamma-Glutamyl Transferase; HDL, High- Density Lipoprotein; HF, Hepatic Fat; HOMA-IR, Homeostatic Model Assessment—Insulin Resistance; IHTG, intrahepatic triglyceride; LAR, Leptin—Adiponectin Ratio; LDL, Low-Density Lipoprotein; NAFLD, nonalcoholic fatty liver disease; SBP, Systolic Blood Pressure*.

**Table 2:** Summary and comparison of baseline characteristics between loss, no change, and gain groups.

**Baseline factors**	**Subjects with paired MRI data (*****N*** **=** **48)**			***P*****-value**	
	**Loss (*N* = 13)**	**No change (*N* = 18)**	**Gain (*N* = 17)**	**All groups**	**Loss vs. Gain**
Hepatic fat %	4.8 (3.7, 7.4)	2.6 (2.0, 3.8)	7.8 (4.5, 15.2)	<0.001	0.16
Subjects with NAFLD	11 (85%)	2 (11%)	8 (47%)	<0.001	0.025
Sex, male	5 (38%)	7 (38.9%)	7 (41%)	0.99	0.88
Age, years	15.2 (12.9, 16.4)	14.6 (13.5, 15.6)	14.6 (13.9, 16.5)	0.61	0.50
Stage of puberty, advanced	10 (77%)	12 (66.7%)	12 (71%)	0.92	0.70
Ethnicity/Race, Hispanic	4 (31%)	3 (16.7%)	7 (41%)	0.26	0.47
Weight (kg)	96.1 (85.2, 114.3)	97.8 (83.2, 108.0)	106.6 (91.4, 113.4)	0.49	0.85
BMI (kg/m^2^)	34.4 (32.8, 41.8)	34.8 (31.8, 38.7)	36.8 (33.7, 39.9)	0.75	0.92
BMI *z*-score	2.4 (2.3, 2.5)	2.3 (2.2, 2.6)	2.5 (2.3, 2.6)	0.84	0.93
Total body fat (%)	45.2 (43.6, 50.7)	44.2 (39.7, 50.3)	46.0 (39.0, 50.1)	0.64	0.48
Waist Circumference (cm)	112 (105, 129)	102.5 (96.0, 119.0)	115.6 (109, 120)	0.22	0.90
SBP (mmHg)	131 (123, 136)	123.5 (120.0, 130.0)	133 (125, 141)	0.086	0.71
DBP (mmHg)	73 (69, 76)	66.0 (65.0, 71.0)	69 (67, 73)	0.014	0.20
Glucose, fasting (mg/dL)	94.0 (86.0, 95.0)	93.0 (88.0, 99.0)	96.0 (86.0, 99.0)	0.72	0.39
Insulin, fasting (mIU/L)	30.3 (26.8, 35.6)	18.5 (15.0, 28.6)	29.0 (20.5, 35.1)	0.12	0.49
HOMA-IR	7.1 (6.2, 7.9)	4.2 (3.5, 6.9)	6.6 (4.3, 7.7)	0.17	0.57
FGF-21 (pg/mL)	209.2 (110.2, 257.7)	156.1 (97.7, 203.1)	131.8 (69.9, 180.5)	0.42	0.23
Adiponectin (ng/mL)	5.8 (3.5, 7.5)	8.5 (5.7, 12.1)	7.1 (5.6, 10.9)	0.087	0.14
Leptin (pg/mL)	55.1 (42.3, 84.2)	51.4 (30.6, 64.0)	55.9 (40.4, 73.0)	0.70	0.69
FAR (pg/ng)	25.9 (16.9, 44.7)	20.8 (11.1, 31.6)	13.7 (11.9, 25.6)	0.12	0.057
LAR (pg/ng)	8.0 (6.8, 24.6)	6.6 (3.7, 9.0)	9.3 (6.4, 10.9)	0.095	0.35
Triglyceride (mg/dL)	124.0 (80.0, 148.0)	89.5 (56.0, 125.0)	110.0 (91.0, 129.0)	0.33	0.95
HDL (mg/dL)	44.0 (38.0, 46.0)	43.0 (38.0, 47.0)	45.0 (41.0, 46.0)	0.79	0.48
FFA (mmol/L)	4.6 (3.9, 5.8)	4.4 (3.1, 5.6)	4.7 (3.6, 6.2)	0.60	0.82
ALT (IU/L)	31.0 (26.0, 45.0)	23.5 (19.0, 34.0)	31.0 (27.0, 37.0)	0.13	0.71
AST (IU/L)	25.0 (21.0, 28.0)	24.0 (20.0, 29.0)	23.0 (21.0, 29.0)	0.99	0.93
GGT (IU/L)	19.0 (16.0, 31.0)	21.0 (18.0, 29.0)	20.0 (18.0, 25.0)	0.98	0.82

*Statistics shown are N (%) or median (Q1, Q3). P values are calculated from Fisher's Exact test for categorical variables, Kruskal–Wallis test for continuous variable comparing all groups, or Wilcoxon Rank-sum test for continuous variables comparing loss and gain groups. ALT, alanine aminotransferase; AST, aspartate aminotransferases; BMI, body mass index; DBP, Diastolic Blood Pressure; FAR, Fibroblast Growth Factor 21–Adiponectin Ratio; FFA, free fatty acid; FGF21, fibroblast growth factor 21; GGT, Gamma-Glutamyl Transferase; HDL, High-Density Lipoprotein; HOMA-IR, Homeostatic Model Assessment—Insulin Resistance; IHTG, intrahepatic triglyceride; LAR, Leptin—Adiponectin Ratio; LDL, Low-Density Lipoprotein; MRI, Magnetic Resonance Imaging; NAFLD, nonalcoholic fatty liver disease; SBP, Systolic Blood Pressure*.

Table 2 summarizes the comparison of baseline characteristics among the loss, no change and gain groups as determined by repeated MRI. To examine how changes in liver fat are associated with baseline FAR and other parameters, comparisons among all three groups, as well as between the loss and gain groups were made. The only pairwise comparison was between loss and gain groups because this was the primary comparison of interest. These two groups were comparable for sex, age, stage of puberty, race/ethnicity, baseline measurements of weight, BMI, BMI *z*-score, percent total body fat, fasting concentrations of glucose, insulin, triglycerides, HDL, free fatty acids, serum ALT, AST, GGT, and LAR levels, and FGF21 and adiponectin concentrations (Table 2). FAR level was higher in the loss group [median (Q1, Q3), 25.9 pg/ng (16.9, 44.7)] compared to gain group [13.7 pg/ng (11.9, 25.6)] (*p* = 0.057) while HOMA-IR and LAR were not different between groups. Although a higher percentage of subjects in the loss group had NAFLD at baseline compared to gain group (85 vs. 47%; *p* = 0.025), the median liver fat percent was not statistically different [4.8% (3.7, 7.4) vs. 7.8% (4.5, 15.2); *p* = 0.16] between the two groups (Table 2).

### Changes in FAR and Liver Fat, and LAR and Liver Fat Are Positively Associated

**Figure 1:**
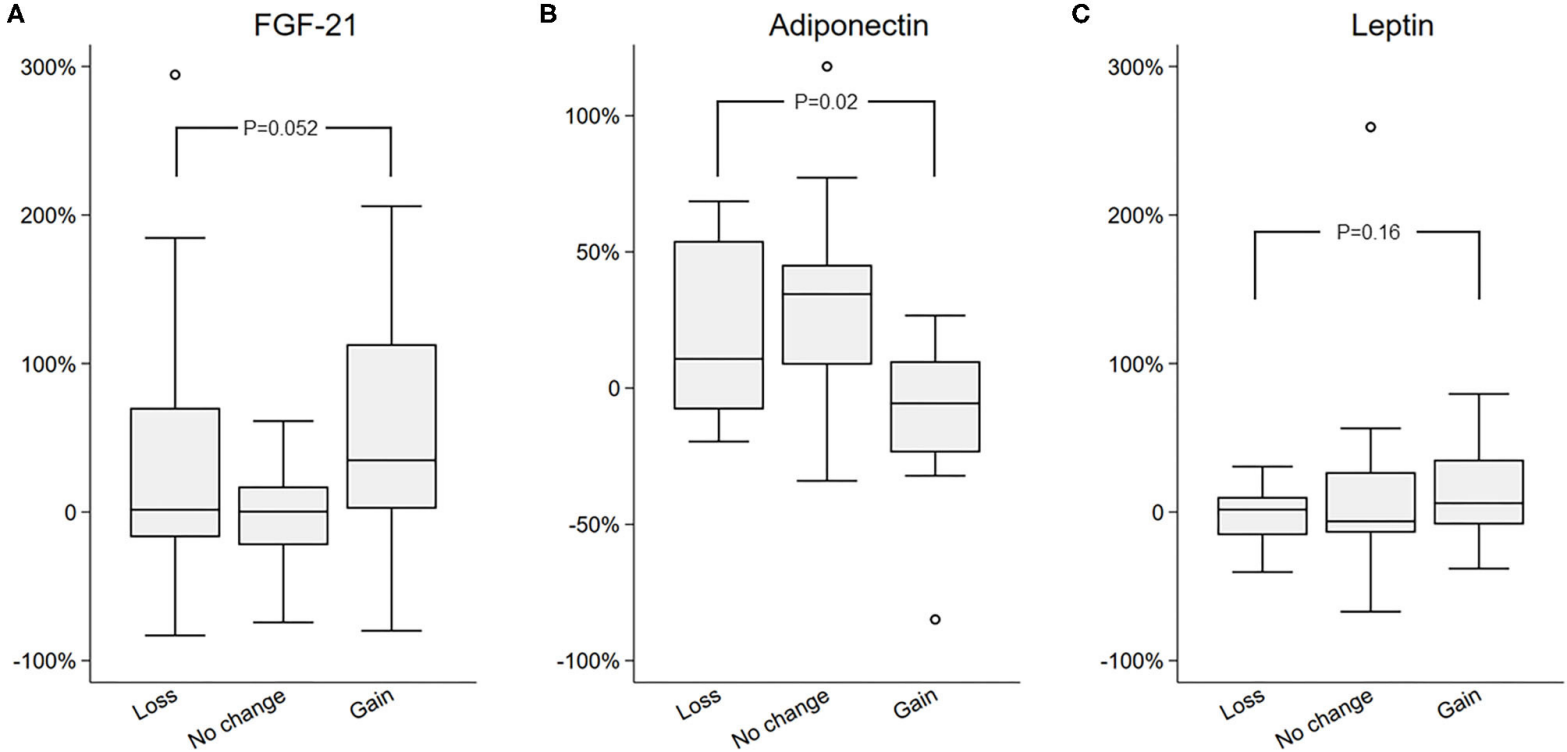
Comparison of percent change in **(A)** FGF21, **(B)** Adiponectin, and **(C)** Leptin in the loss, no change, and gain groups.

**Figure 2:**
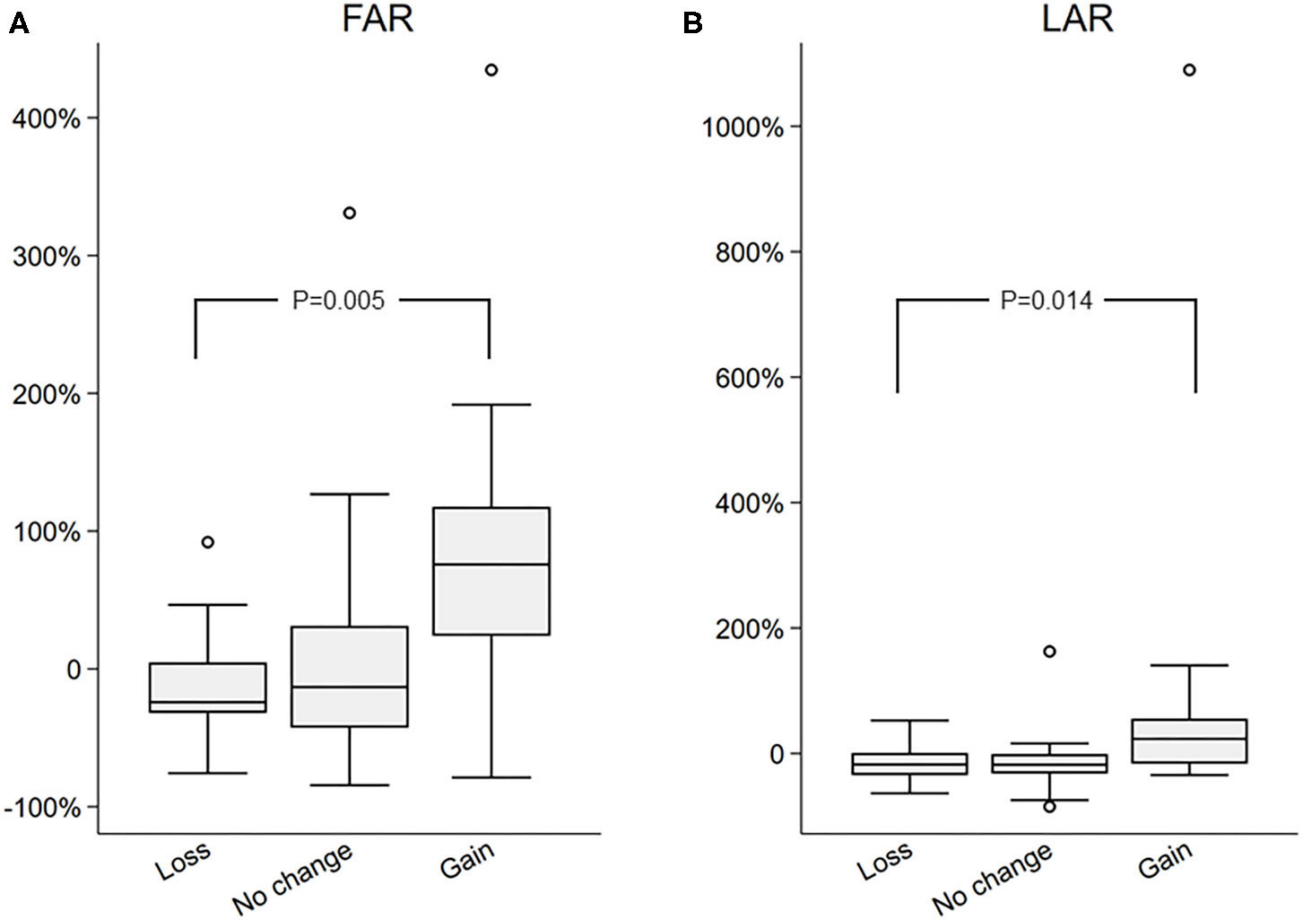
Comparison of percent change in **(A)** FGF21 to Adiponectin Ratio (FAR) and **(B)** Leptin to Adiponectin Ration (LAR) in the loss, no change, and gain groups.

Next, percent changes between two study visits were computed for all biomarkers, and those were compared among the three groups, in order to assess the relationship between change in biomarkers and the changes in the liver fat. Percent change was chosen over absolute change because of the wide between individual variation in biomarkers and non-normal distribution of certain clinical measures, such as FGF21 and FAR. The percent change in FAR was significantly different among groups [−24 (−31, 4) vs. −13 (−42, 31) vs. 75 (23, 117) in the loss vs. no change vs. gain groups, respectively; *p* = 0.005 by Kruskal–Wallis]. Similarly, percent change in LAR was also significantly different among groups [−17 (−34, 1) vs. −17 (−32, 1) vs. 23 (16, 55) in the loss vs. no change vs. gain groups, respectively; *p* = 0.014 by Kruskal–Wallis]. Importantly, there was no difference in the markers of insulin resistance (fasting glucose, insulin, or HOMA-IR) between groups (Table 3). To further appreciate the significant correlation between FAR and LAR and change in liver fat, the percent change of FGF21, adiponectin and leptin (Figure 1), and FAR and LAR (Figure 2) were plotted using a boxplot by the IHTG groups.

**Table 3:** Summary and comparison of percent change in biomarkers between loss, no change, and gain groups.

**Percent (%) change (Δ) in biomarker, %**	**Loss (*N* = 13)**	**No change (*N* = 18)**	**Gain (*N* = 17)**	***P*****-value**	
				**All groups**	**Loss vs. Gain**
%Δ FGF-21	0 (−22, 17)	2 (−17, 70)	35 (2, 113)	0.22	0.052
%Δ Adiponectin	11 (−8, 54)	35 (9, 45)	−6 (−24, 10)	0.005	0.020
%Δ Leptin	2 (−16, 10)	−6 (−14, 27)	6 (−8, 35)	0.39	0.16
%Δ FAR	−24 (−32, 5)	−13 (−43, 31)	76 (24, 118)	0.011	0.005
%Δ LAR	−18 (−35, 1)	−18 (−32, −1)	23 (−16, 56)	0.016	0.014
%Δ Weight	4 (−2, 6)	2 (−1, 5)	6 (4, 8)	0.17	0.31
%Δ BMI *z*-score	−1 (−2, 2)	−1 (−3, 3)	2 (−0, 3)	0.10	0.063
%Δ Percent body fat	−0 (−1, 1)	−2 (−4, 0)	1 (−1, 4)	0.053	0.34
%Δ Waist circumference	1 (0, 2)	1 (0, 6)	2 (1, 6)	0.54	0.30
%Δ Fasting glucose	4 (−6, 12)	−5 (−9, 3)	−2 (−8, 2)	0.35	0.32
%Δ Insulin	−5 (−45, 36)	−14 (−38, 12)	31 (−12, 60)	0.13	0.26
%Δ HOMA-IR	10 (−35, 38)	−8 (−40, 11)	27 (−11, 80)	0.13	0.26
%Δ Triglyceride	2 (−25, 54)	−2 (−37, 8)	23 (2, 59)	0.097	0.39
%Δ HDL	−4 (−8, 6)	−2 (−13, 30)	−5 (−11, 6)	0.81	0.66
%Δ FFA	10 (−29, 36)	2 (−40, 27)	−14 (−23, 10)	0.76	0.52
%Δ ALT	−11 (−13, −6)	4 (−11, 9)	0 (−19, 29)	0.18	0.12
%Δ AST	0 (−6, 4)	0 (−6, 12)	17 (−10, 36)	0.22	0.16
%Δ GGT	0 (−8, 12)	3 (−6, 13)	11 (0, 33)	0.33	0.15

*Statistics shown are median (1st quartile, 3rd quartile). P-values are calculated from Kruskal–Wallis test comparing all groups or Wilcoxon Rank-sum test comparing loss and gain group. ALT, alanine aminotransferase; AST, aspartate aminotransferases; BMI, body mass index; FAR, Fibroblast Growth Factor 21–Adiponectin Ratio; FFA, free fatty acid; FGF21, fibroblast growth factor 21; GGT, Gamma-Glutamyl Transferase; HDL, High- Density Lipoprotein; HOMA-IR, Homeostatic Model Assessment—Insulin Resistance; LAR, Leptin—Adiponectin Ratio; LDL, Low-Density Lipoprotein*.

### Change in FAR, but Not LAR Predicts Final Liver Fat Percent in NAFLD Subjects

**Figure 3:**
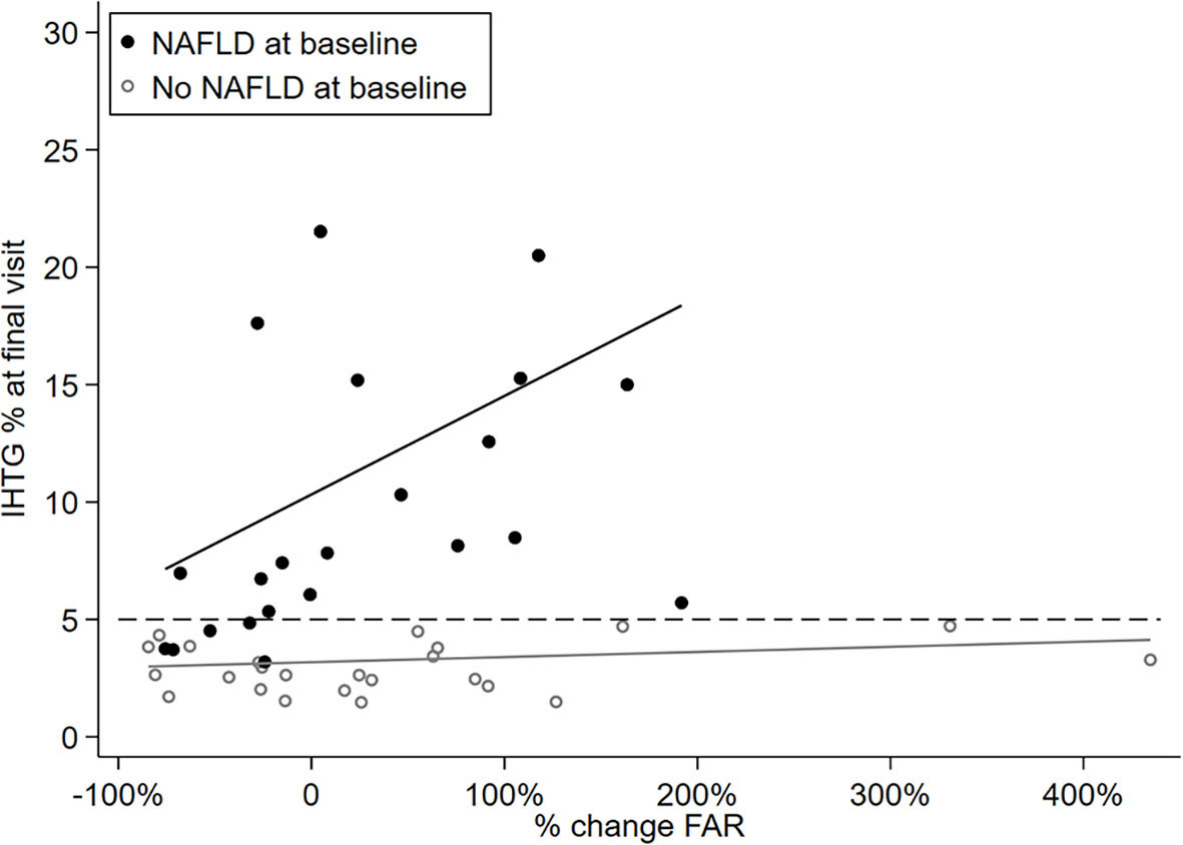
Scatterplot showing linear association between hepatic fat percent at final visit and percent change in the FAR by baseline NALFD status.

Finally, we examined the association between percent change in FAR and the IHTG percent at final visit using a multiple linear regression model including the interactions between percent change in FAR and baseline NAFLD status (Figure 3). Our data showed a significant positive relationship between percent change in FAR and the final IHTG percent in subjects with baseline NAFLD (slope coefficient 6.40, 95% CI 2.23–10.57, *P* = 0.005), but not in those without baseline NAFLD (slope coefficient 0.33, 95% CI −0.25–0.91, *P* = 0.25). Adjusting model for sex and pubertal stage gave similar results (slope coefficient 6.18, 95% CI 1.90–10.47, *P* = 0.007, figure not shown). This suggests that for sex and pubertal stage-similar subjects with baseline NAFLD, the higher value in percent increase in FAR is positively associated with a higher level of liver fat percent at final visit. Using the same multiple linear regression model, we also examined the association between percent change in LAR and the final IHTG percent, but did not find any significant relationship [Slope coefficient: 5.64 (95% CI −1.74, 13.03), *P* = 0.125 for NAFLD; slope coefficient: 0.078 (95% CI −0.27, 0.43) *P* = 0.65 for non-NAFLD].

## Discussion

In this observational study, we investigated the relationship of FGF21, adiponectin, and FAR with IHTG percent in a clinically well-characterized pediatric cohort of pubertal children with obesity participating in a short-term (6-month) lifestyle intervention program. We provided new evidence that FAR associates with NAFLD status even before such a relationship becomes apparent between FGF21 and NAFLD, or adiponectin and NAFLD. We also showed that FAR is positively related to the changes in IHTG percent in subjects with NAFLD even in the absence of a discernable difference in routinely available clinical markers such as weight, BMI-z score, waist circumference, total body fat percent, and serum markers of insulin resistance, liver enzymes, free fatty acid, and lipid profile. Although the FAR was predictive of final liver fat percent in those with NAFLD at baseline, a cause-and-effect relationship cannot be proven in this observational study.

Obesity is generally regarded as an FGF21-resistant state, and weight loss has been shown to be associated with decreasing FGF21 levels (24, 33). Although weight loss is one of the mainstay treatments for the obesity-associated complications such as NAFLD, recent studies focusing on the effect of exercise on NAFLD had shown improved steatosis even when no weight loss was achieved (34–36). Therefore, tracking weight cannot reliably reflect the changes in liver fat. On the other hand, while available evidence seems to suggest that FGF21 patterns may have promising relevance to the assessment of NAFLD status, it alone cannot fully account for differences between no steatosis, simple steatosis, or advanced stages (20, 21, 37, 38). This is mostly related to significant interindividual variations and overlapping values of circulating FGF21 concentrations in lean or obese, and in those with or without NAFLD (20). Furthermore, Dushay et al. reported lower FGF21 levels in patients with NASH compared to simple steatosis (39), and findings of Yan et al. suggest that the lack of a positive relationship between FGF21 levels and steatosis at advanced stages (40). Therefore, FGF21 by itself has limited value as an independent, stand-alone biomarker to diagnose NAFLD or stage the disease. Surprisingly, we have not shown a substantial difference in FGF21 levels among subjects with or without NAFLD in our cohort (Table 1), which could possibly be attributed to different stages of liver disease at baseline in patients with NAFLD in this cohort. Moreover, FGF21 levels were not significantly different among the loss and gain groups matched for baseline liver fat percent.

Adiponectin, as one downstream effector of FGF21, is believed to antagonize excess hepatic lipid accumulation through stimulation of fatty acid oxidation and inhibition of fatty acid synthase activity in the liver (16, 41, 42). Interestingly, the physiological relationship between these two hormones appear to be dissociated under pathological conditions such as insulin resistance and NAFLD (17) For instance, epidemiological studies in adults showed that lower baseline serum adiponectin (40, 43, 44) and higher baseline serum FGF21 levels (38) are independent predictors of NAFLD development. However, pharmacological doses of FGF21 treatment have been shown to restore this impaired relationship even in human subjects with diabetes and NAFLD, as demonstrated in a small number of clinical trials (25–27). In a randomized, double-blinded, placebo-controlled study, Charles et al. showed a positive dose-dependent relationship between PEGylated FGF21 analog treatment and serum adiponectin levels in obese patients with type 2 diabetes and NAFLD while no significant change in HOMA-IR was observed (27) Their findings provide direct evidence that FGF21 has a substantial role in the regulation of circulating adiponectin concentrations even in the absence of a change in insulin resistance state. Although the NAFLD status was only indirectly assessed, the authors showed improvements in NAFLD-associated biomarkers, which, in part, was attributed to increased adiponectin levels (27). Rodent studies have also demonstrated a positive relationship between FGF21 and adiponectin (14, 17), while also shown that the beneficial effects of FGF21 on steatosis are ablated in adiponectin knock-out mice (12, 45). These reports provide the basis that FGF21-Adiponectin Ratio (FAR) could be a promising tool to detect the presence of steatosis and even monitor the change in liver fat given the inverse relationship between FGF21 and adiponectin levels in the circulation. Our findings support this notion, as FAR in our cohort was associated with the NAFLD status even before such association was observed between FGF21 or adiponectin as individual hormones. Besides, percent change in FAR was also related to the liver fat percent at the final visit in those with NAFLD at baseline.

The importance of these findings and the utility of FAR in clinical practice requires further investigations. Current study is not designed to identify the diagnostic role of FAR as a biomarker. Although FAR level was different between subjects with or without NAFLD, there is no recommended pediatric cut-point in the literature to test the sensitivity or specificity of our results. Also, how FAR relates to other clinical markers (e.g., HOMA-IR) and the effect of such interaction on the NAFLD outcomes are yet to be determined.

There are a few limitations to our study. Although FGF21 is a potent stimulus for adiponectin secretion, it is not the only one. Proinflammatory cytokines and oxidative stress, often upregulated in obesity, have direct regulatory roles on adiponectin secretion. Since there were no significant changes in BMI z-scores throughout the follow-up period among the loss, no change, or gain groups, it allowed us to evaluate associations of liver fat and serum hormones in a weight-independent manner. Also, a lack of difference in HOMA-IR among groups further suggests that the change in FAR is likely due to the regulatory effect of FGF21 on adiponectin and not an effect of insulin resistance on the latter. However, it is acknowledged that HOMA-IR is only an indirect measure of insulin resistance and cannot reliably assess differential insulin resistance in different body sites as do the clamps studies. Another potential limitation is that the MRI method used in this study did not assess presence of fibrosis, which could have affected the FGF21 and adiponectin levels. That said, given the characteristics of the cohort (young age, no known other risk factors such as diabetes, etc.) this may have had negligible relevance. Although we did not have histological data to compare, we strongly believe that one-percent point change in liver fat is a remarkable change to produce changes in liver histology as suggested by Patel et al. (32). Furthermore, we explored different cut off points, ranging from ± 1–± 1.5% with 0.1% increments, to define these three groups (loss, no change, gain) and the resulting distribution of percent change in liver fat content between groups was similar, while the ± 1% point provided the most balanced group assignment (Supplementary Table 4). The predictive value of the FAR might, in theory, be improved by adding more biomarker variables or methods, such as transient elastography with controlled attenuated parameter (Fibroscan®, EchoSens, Paris). FibroScan is being used with increasing frequency in adults for the diagnosis and monitoring of various liver conditions. However, its utility in the pediatric population has been under-explored. Furthermore, FibroScan provides a semi-quantitative and static assessment of the liver fat, and the diagnostic cut-off points for the controlled attenuated parameter in children are currently missing (in fact, elastography measurements to assess fibrosis in children are not fully established). Thus, longitudinal studies to compare performances of the FAR and FibroScan findings in the diagnosis and monitoring of NAFLD would be helpful only when norms are established. Finally, the lack of such an association between the FAR and change in liver fat in subjects without NAFLD at baseline requires further investigation. Unfortunately, we were unable to compare between the groups defined by a change in NAFLD status due to the small number of subjects flipping NAFLD status at the end of the study period (five children with NAFLD at baseline had resolution of the NAFLD status at 6-months, and two subjects who did not have NAFLD at baseline had developed NAFLD at the final visit). Study duration was limited to 6-months to minimize the effect of pubertal progression on results, and 6-months was previously shown to be sufficient time to achieve a meaningful decline in the weight of the subjects participating in a weight management therapy primarily focusing on lifestyle interventions.

In conclusion, the FGF21-Adiponectin Ratio was associated with NAFLD status, and there was a positive correlation between FAR and final liver fat, even after controlling for sex and pubertal stage, in those with NAFLD at baseline as determined by repeated MRI scans before and after a 6-month lifestyle intervention. These findings suggest that the monitoring the change in FAR could be a promising clinical tool to help detect a clinically meaningful change in liver fat independent of or in combination with anthropometrics or routinely used biomarkers (e.g., HOMA-IR, ALT).

## Data Availability Statement

The raw data supporting the conclusions of this article will be made available by the authors, without undue reservation, to any qualified researcher.

## Ethics Statement

The studies involving human participants were reviewed and approved by Institutional Review Board at the University of Arkansas for Medical Sciences. Written informed consent to participate in this study was provided by the participants' legal guardian/next of kin.

## Author Contributions

The authors' responsibilities were as follows: ET and SB designed the research. ET conducted the study. ET, XO, KMe, HL, KMa, RB, ED, JO, EB, SA, and JD processed and interpreted the clinical and imaging data. SB performed the statistical analyses. ET and SB wrote the manuscript. ET had primary responsibility for final content and edits. All authors read and approve the final manuscript.

## Conflict of Interest

The authors declare that the research was conducted in the absence of any commercial or financial relationships that could be construed as a potential conflict of interest.

## Abbreviations

HbA1c: hemoglobin A1c
ALT: alanine aminotransferase
AST: aspartate aminotransferases
BMI: body mass index
DXA: dual-energy X-ray absorptiometry
FAR: Fibroblast Growth Factor 21–Adiponectin Ratio
FFA: free fatty acids
FGF21: fibroblast growth factor 21
GGT: Gamma-Glutamyl Transferase
IHTG: intrahepatic triglycerides
IR: insulin resistance
MRI: magnetic resonance imaging
NAFLD: non-alcoholic fatty liver disease.
