# Supplementary material for: Fibroblast Growth Factor-21 to Adiponectin Ratio: A Potential Biomarker to Monitor Liver Fat in Children With Obesity
Source: Front Endocrinol (Lausanne). 2020 Sep 17;11:654. doi: 10.3389/fendo.2020.00654 (PMC7533567; doi:10.3389/fendo.2020.00654)
Supplement: Supplementary file 1 [file Data_Sheet_1.PDF]

frontiers

Supplementary Material

Supplementary Table 1: Comparison of baseline markers by sex (via two-sample t-test).
Abbreviations: ALT, alanine aminotransferase; AST, aspartate aminotransferases; BMI, body mass index; FAR, Fibroblast Growth Factor 21 - Adiponectin Ratio; FFA, free fatty acid; FGF21, fibroblast growth factor 21; GGT, Gamma-Glutamyl Transferase; HDL, High- Density Lipoprotein; HOMA-IR, Homeostatic Model Assessment – Insulin Resistance; LAR, Leptin to Adiponectin Ratio; MRI, magnetic resonance imaging.; SD, standard deviation.

Factor (mean, SD)	Female	Male	p-value
N	34	26	
Weight (kg)	100.7 (21.8)	102.6 (22.5)	0.75
BMI (kg/m^2)	38.1 (7.0)	36.1 (5.2)	0.22
BMI Z-score	2.4 (0.3)	2.5 (0.3)	0.16
Percent Body Fat (%)	47.3 (5.4)	41.2 (5.9)	<0.001
Waist Circumference (cm)	113.7 (15.1)	112.2 (14.0)	0.69
Systolic Blood Pressure (mmHg)	126.4 (9.7)	129.5 (10.2)	0.23
Diastolic Blood Pressure (mmHg)	70.9 (6.1)	69.3 (4.4)	0.24
Fasting Glucose (mg/dL)	92.7 (10.7)	94.8 (7.5)	0.39
Fasting Insulin (uIU/mL)	31.1 (18.5)	26.9 (16.3)	0.36
HOMA-IR (arbitrary unit)	7.2 (4.5)	6.3 (3.8)	0.43
Fasting FGF-21 (pg/mL)	150.8 (104.5)	163.6 (105.6)	0.64
Fasting Adiponectin (ng/mL))	7.3 (2.8)	8.1 (4.5)	0.41
FAR (pg/ng)	28.2 (37.0)	22.2 (12.0)	0.44
LAR (pg/ng)	12.7 (8.5)	6.5 (4.1)	0.001
Fasting TG (mg/dL)	108.8 (61.3)	111.2 (52.3)	0.87
Fasting HDL (mg/dL)	45.2 (8.5)	42.1 (7.8)	0.14
FFA (mmol/L)	4.4 (1.9)	5.1 (2.1)	0.23
Fasting Leptin (pg/mL)	75.5 (34.1)	40.1 (18.6)	<0.001
ALT (IU/L)	27.5 (9.3)	38.8 (16.2)	0.001
AST (IU/L)	23.4 (5.5)	34.2 (16.5)	<0.001
GGT (IU/L)	20.8 (7.2)	29.0 (12.3)	0.002

Supplementary Table 2: Comparison of baseline marker by puberty (via two-sample t-test)
Abbreviations: ALT, alanine aminotransferase; AST, aspartate aminotransferases; BMI, body mass index; FAR, Fibroblast Growth Factor 21 - Adiponectin Ratio; FFA, free fatty acid; FGF21, fibroblast growth factor 21; GGT, Gamma-Glutamyl Transferase; HDL, High- Density Lipoprotein; HOMA-IR, Homeostatic Model Assessment – Insulin Resistance; LAR, Leptin to Adiponectin Ratio; MRI, magnetic resonance imaging.; SD, standard deviation.

Factor (mean, SD)	Early stages (Tanner stage II and III)	Late stages (Tanner stage IV and V)	p-value
N	17	43	
Weight (kg)	92.7 (20.1)	105.0 (21.8)	0.050
BMI (kg/m^2)	34.7 (4.9)	38.2 (6.6)	0.052
BMI Z-score	2.5 (0.3)	2.4 (0.3)	0.69
Percent Body Fat (%)	42.4 (6.5)	45.6 (6.2)	0.082
Waist Circumference (cm)	108.6 (13.0)	114.8 (14.9)	0.14
Systolic Blood Pressure (mmHg)	127.6 (10.6)	127.8 (9.8)	0.94
Diastolic Blood Pressure (mmHg)	68.8 (4.0)	70.8 (5.9)	0.22
Fasting Glucose (mg/dL)	93.0 (6.5)	93.8 (10.4)	0.76
Fasting Insulin (uIU/mL)	27.3 (23.7)	30.1 (14.7)	0.59
HOMA-IR (arbitrary unit)	6.3 (5.3)	7.0 (3.7)	0.54
Fasting FGF-21 (pg/mL)	189.0 (113.7)	144.0 (99.2)	0.14
Fasting Adiponectin (ng/mL))	8.7 (5.1)	7.2 (2.9)	0.17
FAR (pg/ng)	25.0 (13.4)	25.9 (33.3)	0.92
LAR (pg/ng)	6.2 (3.9)	11.5 (8.2)	0.015
Fasting TG (mg/dL)	108.6 (51.9)	110.3 (59.6)	0.92
Fasting HDL (mg/dL)	42.7 (7.8)	44.3 (8.5)	0.50
FFA (mmol/L)	5.6 (2.5)	4.4 (1.8)	0.043
Fasting Leptin (pg/mL)	39.6 (19.1)	68.3 (34.4)	0.003
ALT (IU/L)	32.4 (9.5)	32.4 (15.3)	>0.99
AST (IU/L)	30.2 (11.2)	27.2 (13.3)	0.42
GGT (IU/L)	22.7 (9.8)	25.0 (10.8)	0.45

Supplementary Table 3: Correlation data between percent change in intrahepatic triglycerides and other biomarkers.

Abbreviations: ALT, alanine aminotransferase; AST, aspartate aminotransferases; BMI, body mass index; FAR, Fibroblast Growth Factor 21 - Adiponectin Ratio; FFA, free fatty acid; FGF21, fibroblast growth factor 21; GGT, Gamma-Glutamyl Transferase; HDL, High- Density Lipoprotein; HOMA-IR, Homeostatic Model Assessment – Insulin Resistance; MRI, magnetic resonance imaging.

Variables in the same order as in Table 2		Percent Change in IHTG between two MRI		
		All (N=58)	Baseline NAFLD Negative (N=35)	Baseline NAFLD Positive (N=23)
% change_FGF21	Correlation	0.1444	0.1248	0.1734
	P-value	0.3327	0.5189	0.4913
	N	47	29	18
% change_Adiponectin	Correlation	-0.478	-0.4961	-0.4606
	P-value	0.0007	0.0062	0.0544
	N	47	29	18
% change_FAR	Correlation	0.5288	0.6016	0.344
	P-value	0.0001	0.0006	0.1622
	N	47	29	18
% change_weight	Correlation	0.3178	0.4836	-0.0085
	P-value	0.0277	0.0079	0.9726
	N	48	29	19
% change_BMI-z	Correlation	0.3398	0.4056	0.1456
	P-value	0.0181	0.029	0.552
	N	48	29	19
% change_Percent Body Fat	Correlation	0.4256	0.4002	0.5285
	P-value	0.0026	0.0315	0.02
	N	48	29	19
% change_waist circumference	Correlation	0.1559	0.2425	-0.0424
	P-value	0.29	0.205	0.8632
	N	48	29	19
% change_glucose	Correlation	-0.144	-0.0309	-0.6035
	P-value	0.3454	0.8783	0.008
	N	45	27	18
% change_insulin	Correlation	0.05	0.109	0.0284
	P-value	0.7443	0.5883	0.9109
	N	45	27	18
% change_HOMA-IR	Correlation	0.0337	0.1541	-0.0609
	P-value	0.826	0.4429	0.8102
	N	45	27	18
% change_Leptin	Correlation	0.1569	0.3188	0.0091
	P-value	0.2922	0.0919	0.9716
	N	47	29	18
% change_Triglyceride	Correlation	0.0213	-0.073	0.351
	P-value	0.8896	0.7176	0.1533
	N	45	27	18
% change_HDL	Correlation	-0.1615	-0.1891	-0.093
	P-value	0.2893	0.3447	0.7135
	N	45	27	18
% change_FFA	Correlation	0.016	0.0763	-0.0686
	P-value	0.9139	0.6941	0.7804
	N	48	29	19
% change_ALT	Correlation	0.1635	0.0331	0.5039
	P-value	0.2832	0.8698	0.033
	N	45	27	18
% change_AST	Correlation	0.2377	0.2356	0.4251
	P-value	0.1159	0.2368	0.0786
	N	45	27	18
% change_GGT	Correlation	0.0936	0.058	0.2526
	P-value	0.541	0.7738	0.3119
	N	45	27	18

Supplementary Table 4: Comparison of relative reduction or increment in the mean intrahepatic triglyceride (IHTG) percent based on select cut-points to determine the absolute change in IHTG percent needed to generate the most balanced groups to test clinical significance.

		**Relative change in IHTG Percent**		
	**N**	**Mean**	**Standard deviation**	**Difference between loss and gain**
**Cut off=-1.5 and 1.5 %**				
Loss	10	-0.311	0.112	1.061
No change	23	0.025	0.312	
Gain	15	0.75	0.613	
**Cut off=-1.4 and 1.4 %**				
Loss	11	-0.308	0.107	1.058
No change	22	0.039	0.312	
Gain	15	0.75	0.613	
**Cut off=-1.3 and 1.3 %**				
Loss	12	-0.311	0.102	1.061
No change	21	0.058	0.307	
Gain	15	0.75	0.613	
**Cut off=-1.2 and 1.2 %**				
Loss	12	-0.311	0.102	1.032
No change	20	0.047	0.311	
Gain	16	0.721	0.603	
**Cut off=-1.1 and 1.1 %**				
Loss	13	-0.303	0.103	1.024
No change	19	0.06	0.313	
Gain	16	0.721	0.603	
**Cut off=-1 and 1 %**				
Loss	13	-0.303	0.103	1.042
No change	18	0.006	0.217	
Gain	17	0.739	0.589	
